# Vitamin D Supplementation Modulates Platelet-Mediated Inflammation in Subjects With Type 2 Diabetes: A Randomized, Double-Blind, Placebo-Controlled Trial

**DOI:** 10.3389/fimmu.2022.869591

**Published:** 2022-05-26

**Authors:** Ebin Johny, Aishwarya Jala, Bishamber Nath, Md Jahangir Alam, Indra Kuladhipati, Rupam Das, Roshan M. Borkar, Ramu Adela

**Affiliations:** ^1^ Department of Pharmacy Practice, National Institute of Pharmaceutical Education and Research, Guwahati, India; ^2^ Department of Pharmaceutical Analysis, National Institute of Pharmaceutical Education and Research, Guwahati, India; ^3^ Department of Biotechnology, National Institute of Pharmaceutical Education and Research, Guwahati, India; ^4^ Downtown Hospital, Guwahati, India

**Keywords:** type 2 diabetes, vitamin D, platelet, inflammation, oxidative stress

## Abstract

**Background:**

Recently, our group identified increased platelet-mediated inflammation in type 2 diabetes (T2DM) patients, and it is a well-established risk factor for diabetes complications, particularly for the development of cardiovascular diseases (CVD). Furthermore, vitamin D is reported to play an important role in the modulation of platelet hyperactivity and immune function, although the effect of vitamin D on platelet-mediated inflammation is not well studied. Hence, we aimed to investigate the effect of vitamin D supplementation on platelet-mediated inflammation in T2DM patients.

**Methods:**

After screening a total of 201 subjects, our randomized, double-blind, placebo-controlled trial included 59 vitamin-D-deficient T2DM subjects, and the participants were randomly assigned to placebo (*n* = 29) or vitamin D3 (*n* = 30) for 6 months. Serum vitamin D metabolite levels, immunome profiling, platelet activation, and platelet–immune cell aggregate formation were measured at baseline and at the end of the study. Similarly, the serum levels of inflammatory cytokines/chemokines were assessed by a multiplex assay.

**Results:**

Six months of vitamin D supplementation increases the serum vitamin D3 and total 25(OH)D levels from the baseline (*p* < 0.05). Vitamin D supplementation does not improve glycemic control, and no significant difference was observed in immune cells. However, platelet activation and platelet immune cell aggregates were altered after the vitamin D intervention (*p* < 0.05). Moreover, vitamin D reduces the serum levels of IL-18, TNF-α, IFN-γ, CXCL-10, CXCL-12, CCL-2, CCL-5, CCL-11, and PF-4 levels compared to the baseline levels (*p* < 0.05). Our *ex vivo* experiment confirms that a sufficient circulating level of vitamin D reduces platelet activation and platelet intracellular reactive oxygen species.

**Conclusion:**

Our study results provide evidence that vitamin D supportive therapy may help to reduce or prevent the disease progression and cardiovascular risk in T2DM patients by suppressing oxidative stress and platelet-mediated inflammation.

**Clinical Trial Registration:**

Clinical Trial Registry of India: CTRI/2019/01/016921.

## Introduction

Type 2 diabetes (T2DM) is a chronic metabolic disorder characterized by elevated blood glucose levels and insulin resistance. Diabetes remains a critical public health concern worldwide, with an estimated 537 million adults suffering from the disease and with T2DM accounting for 90% of all diabetes cases (1). Diabetes affects multiple organs, including the heart, muscle, skin, eyes, brain, and kidneys, and causes microvascular and macrovascular complications. Diabetic subjects exhibit a two- to four-fold more significant risk of developing cardiovascular disorders when compared to non-diabetic individuals (2). Insulin resistance and hyperglycemia together trigger inflammation, oxidative stress, and endothelial dysfunction, contributing to the development of cardiovascular diseases in type 2 diabetes patients. Platelet activation and platelet-mediated inflammation are other critical factors implicated in the pathogenesis of coronary artery disease in type 2 diabetes patients. Recently, our group has shown that hyperglycemia in type 2 diabetes causes platelet activation; aggregation further contributes to systemic inflammation and the complexity of both type 2 diabetes and the development of cardiovascular diseases (3).

Vitamin D is classified as a secosteroid and exists in two forms, *i*.*e*., ergocalciferol (vitamin D2) and cholecalciferol (vitamin D3). Our previous article reported that lower vitamin D levels are associated with T2DM with coronary artery disease (2). Furthermore, vitamin D has recently gained prominence as a diabetes risk modifier as vitamin D supplementation has reduced the incidence of T2DM and improved glycemic control, owing to increased insulin production (4), decreased insulin resistance (5), and reduced inflammation (6). Several observational studies have also reported an inverse relationship between platelet activation and vitamin D levels in different disease conditions (7, 8). Recently, Sultan et al. reported that lower vitamin D levels significantly contribute to increased platelet aggregation in type 2 diabetes patients (9). However, there is a lack of evidence on the effect of vitamin D supplementation on platelet-mediated inflammation in type 2 diabetes. This study aimed to determine the effect of vitamin D supplementation on vitamin-D-deficient type 2 diabetes patients, focusing on platelet activation and platelet-mediated systemic inflammation.

## Methods

### Study Design

The study was a single-center, randomized, double-blind, placebo-controlled study, and the participants were recruited from the outpatient unit of the departments of diabetes and cardiology at Downtown Hospital, Guwahati, Assam, India. The study was approved by Downtown Hospital Ethical Committee with approval number EC/DTH/CT/2018/10 and registered in the Clinical Trial Registry of India (CTRI) with registration number CTRI/2019/01/016921. The study was conducted from July 2019 to January 2021. The clinical trial was conducted following the principles outlined in the Declaration of Helsinki and the institutional and ethical standards. The individuals supplied written informed consent at the screening visit, and data on clinical history, physical examinations, and blood samples were obtained to confirm the eligibility criteria. The study design is shown in **Supplementary Figure S1A**.

### Study Population

**Figure 1:**
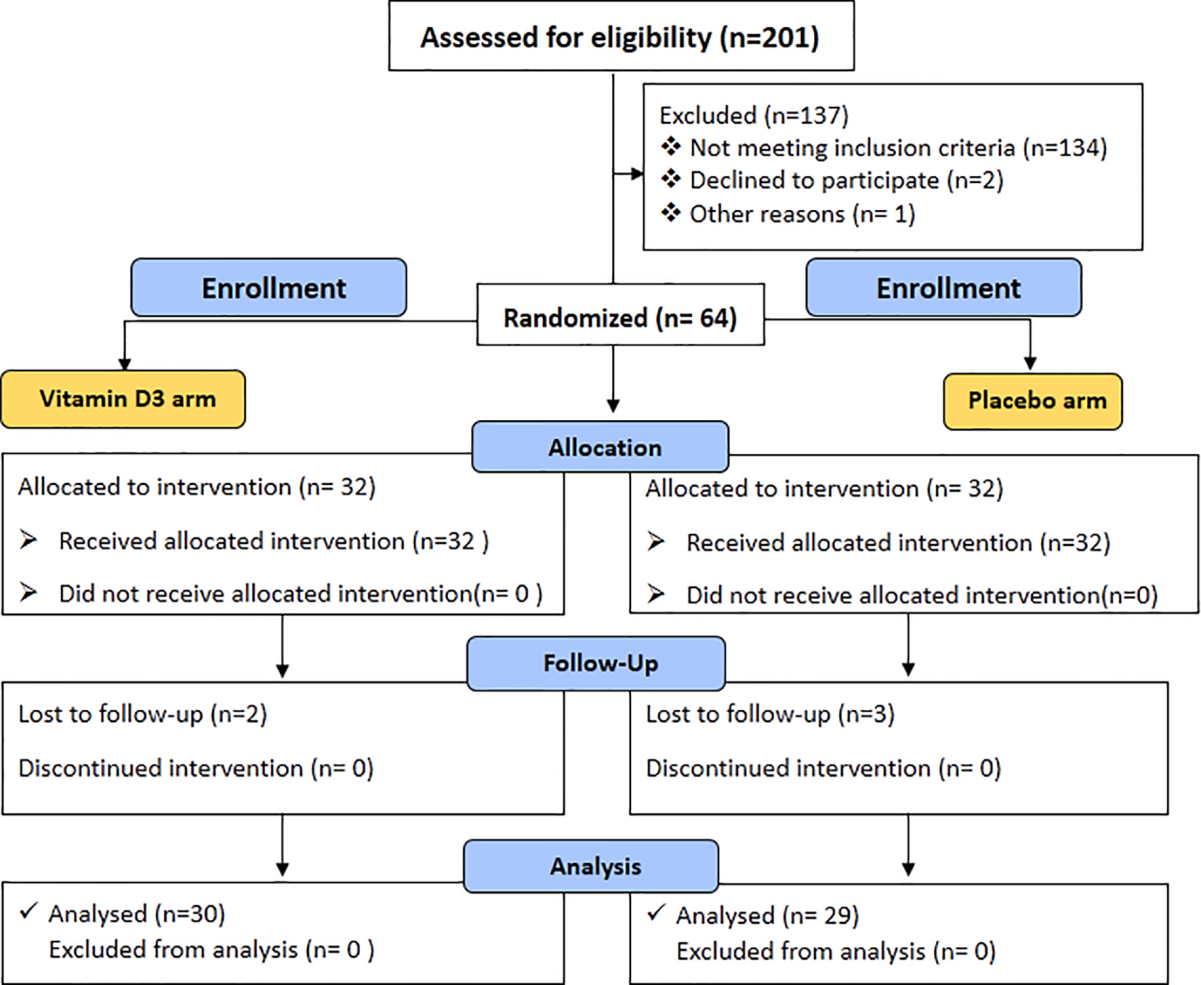
Consort 2010 flow diagram.

The study includes uncontrolled type 2 diabetes subjects with HbA1c ≥7% as defined by the American Diabetes Association (10, 11). Another inclusion criterion was subjects aged between 35 to 65 years with vitamin D deficiency [total 25(OH) D ≤20 ng/ml] at the baseline visit. The exclusion criteria were subjects who had a prior treatment with vitamin D, clinical or laboratory evidence of chronic disease of liver failure, renal failure, type 1 diabetes, cancer, and thyroid disease, and pregnancy. The overall flow chart of the study is shown in **Figure 1**.

### Randomization and Intervention

With computer-generated randomization codes, the volunteers were randomly assigned to either the placebo block or the vitamin D3 block. The vitamin D3 group received 60,000 IU cholecalciferol/week for the initial 3 months as a management dose, followed by 60,000 IU/month for 3 months as a maintenance dose. The placebo group received a matching placebo (composed of starch powder) similar to vitamin D. After completing 6 months of intervention, data on clinical history, physical examinations, and blood samples were obtained. The vitamin D dosage pattern is shown in **Supplementary Figure S1B**.

### Measurement of Clinical and Biochemical Parameters

Standardized methodologies were used to assess the height, weight, and hip and waist circumference. The body mass index (BMI) was determined by dividing the weight in kilograms (kg) by the square of the height in meters (m^2^). The waist-to-hip ratio was obtained by dividing the waist circumference by the hip circumference. Blood pressure (BP) was measured in triplicate with Omron HEM automated BP monitor. The Xpress A1C analyzer (Accurex Biomedical Pvt. Ltd) was used to determine the glycated hemoglobin (HbA1c) level. The serum levels of total cholesterol (TC), triglycerides (TG), high-density lipoprotein (HDL), low-density lipoprotein (LDL), creatinine, and uric acid were analyzed using Erba Chem 7 Biochemistry Analyser (Transasia Bio-Medicals, India). The urinary creatinine levels were measured by a colorimetric detection method (catalog number 40620, Accurex Biomedical). Human insulin levels were measured by using the ELISA kit (catalog number KBH0001, Krishgen Biosystems). HOMA-IR values were calculated by using the following formula: fasting blood sugar (FBS, mmol/L) × fasting insulin (mIU)/22.5.

### Sample Preparation and Quantification of Vitamin D Metabolites by LC–MS/MS

The stock solutions of vitamin D3, vitamin D2, and their metabolites [25(OH)D3, 1,25(OH)2D3, 25(OH)D2, and 1,25(OH)2D2] were prepared in ethanol. Furthermore, the working solution for the calibration curve (5–200 ng/ml) was prepared in methanol from the primary stock solutions. Similarly, an internal standard (IS) was prepared, *i*.*e*., dihydrotachysterol of 50 ng/ml.

The extraction of vitamin D metabolites from serum samples was performed following the protocol described in our previously reported studies (2, 12). Briefly, liquid–liquid extraction technique was used to extract vitamin D metabolites from serum samples. A mixture of hexane/heptane/acetone—in the ratio of 45:40:15—was used as an extraction solvent. To 100 µl of serum sample, 10 µl of IS was added, followed by the addition of 1 ml extraction solvent. The mixture was vortexed thoroughly and placed in a shaker for 10 min. The samples were centrifuged at 6,000 rpm for 10 min at 4°C. The resulting supernatant was collected and dried under vacuum using an Eppendorf concentrator plus a centrifuge concentrator. The concentrated sample is reconstituted in 100 µl of methanol and subjected to liquid chromatography–mass spectrometry/mass spectrometry (LC–MS/MS) analysis. The details of the method, validation data (**Supplementary Table S1**), and LC–MS/MS chromatograms for vitamin D (**Supplementary Figure S2**) and its metabolite are provided in the supplementary file.

### Measurement of Vitamin-D-Binding Protein

The human vitamin-D-binding protein (VDBP) was measured in the serum samples using a standard enzyme-linked immunosorbent assay protocol according to the manufacturer’s instructions (catalog number DVDBP0B, R&D systems).

### Flow Cytometry Analysis

The flow cytometry analysis was performed to measure platelet activation, detect immunome profiling, and perform platelet–immune cell aggregate analysis following the protocol described in our previously reported study (3). Briefly, blood was collected by venepuncture in acid citrate dextrose (BD Vacutainer^®^) tubes for all the flow cytometry experiments and processed for analysis within 2 h of blood collection.

### Measurement of Platelet Activation

Platelet-rich plasma (PRP) was isolated, and 25 µl of PRP was incubated with platelet activation markers PAC-1-FITC (BD, catalog number 340507) and CD62P)-APC (BD, catalog number 550888) for 20 min at room temperature. The platelets were washed with phosphate-buffered saline (PBS), resuspended in PBS, and analyzed in Attune NxT flow cytometer (Thermo Fisher Scientific, Singapore). The gating strategy for measuring the percent of platelet activation is described in **Supplementary Figure S3**.

### Measurement of Platelet Factor 4

Platelet activation marker platelet factor 4 (PF-4) was measured in the serum samples using the ELISA method (catalog number K12-4574), according to the manufacturer’s instructions.

### Immunome Profiling and Measurement of Platelet–Immune CellAggregate Formation

Immunome profiling and platelet–immune cell aggregates were measured, as mentioned in our previous article (3). Briefly, whole blood was lysed, and cells were suspended in PBS with 1% w/v bovine serum albumin. Furthermore, the cells were incubated with respective antibodies for 20 min at room temperature. The cells were washed with PBS and analyzed by a flow cytometer. For compensation purposes, unstained and single antibody-stained cells were used. Work was divided into three panels. Each panel consists of one stained and unstained sample. Leucocytes were identified based on the forward scatter (FSC) and side scatter characteristics, and the same was confirmed using specific cell surface markers. Monocytes were identified based on FSC *vs*. SSC, were confirmed by CD14 APC Cy 7, and were further subdivided based on their differential expression of CD14 and CD16 into classical (CD14++ CD16-), intermediate (CD14++ CD16+), and non-classical (CD14+ CD16++) monocytes. Similarly, T cells were confirmed by their surface expression of CD3 from lymphocyte populations and further gated to obtain CD4 T cells (T-helper cells, CD3+ CD4+), CD8 T cells (cytotoxic T cells, CD3+ CD8+), and natural killer T cells (CD3+ CD56+). From the CD3-ve cells, natural killer cells (CD3- CD56+) were also identified. Dendritic cells (lineage–HLA DR+) were identified from WBC populations and were further subdivided into myeloid (HLA DR+ CD11C+) and plasmacytoid (HLA DR+ CD123+) dendritic cells. Platelet surface marker CD41a–PECY5 (BD, catalog number 559768) was included in each panel to detect platelet–immune cell interaction (3). The gating strategy for immunome profiling and detection of platelet–immune cell aggregation is described in **Supplementary Figures S4**, **S5**. The fluorescence minus one method for identifying platelet–immune cell aggregates is demonstrated in **Supplementary Figure S6**. AttuneTM NxT software v3.1.2 was used to perform the flow cytometry data analysis.

### Measurement of Urinary 11-Dehydrothromboxane B2 by LC–MS/MS

Urinary 11-dehydrothromboxane B2 was measured by LC–MS/MS method. The extraction of 11-dehydroythromboxane B2 from urine samples was performed following solid-phase extraction (13). The separation of 11-dehydroythromboxane B2 and IS from endogenous substances was achieved using ZORBAX Eclipse Plus C18 Rapid Resolution HD (2.1 × 50 mm, 1.8 μm) and mobile phase consisting of a mixture of 2 mM ammonium formate with 0.1% formic acid (A) and methanol with 0.1% formic acid (B) in a gradient program mode. The details of the method, method validation data (**Supplementary Table S2**), and LC–MS/MS chromatograms for 11-dehydroythromboxane B2 (**Supplementary Figure S7**) are provided in the supplementary file.

### Measurement of Circulatory Inflammatory Markers

Circulating levels of cytokines and chemokines, *i*.*e*., IL-1β, IL-2, IL-4, IL-5, IL-6, IL-8, IL-12p70, IL-13, IL-18, TNFα, IFN-γ, GM-CSF, CXCL-1, CXCL-10, CXCL-12, CCL-2, CCL-3, CCL-4, CCL-5, and CCL-11, were measured using Invitrogen ProcartaPlex Hu Th1/Th2/multiplex immunoassay kit (catalog number EPX200-12173-901). Stored serum samples were thawed and centrifuged (10,000 rpm) to remove the debris. All experiments were performed according to manufacturer’s protocol using an automated microplate washer (Bio-Rad) and analyzed using Bio-Plex 200 systems (Bio-Rad). Four of the 20 cytokines analyzed (IL-2, IL-5, IL-6, and GM-CSF) were omitted from further analysis because either more than 95% of the analyte concentration was below the lowest standard or its maximal fluorescence intensity value was near the background. The manufacturer provided standards for all cytokines and chemokines. A serum sample of 25 µl was used, and 100 bead events/bead region was acquired. The mean florescent intensity was measured using Bio-Plex manager software, version 6.2. All samples were measured in singlet, whereas blank and standards were measured in duplicate.

### Measurement of Oxidative Stress Markers and Nitric Oxide Levels

To find the effect of vitamin D supplementation on oxidative stress, we have measured the circulatory superoxide dismutase (SOD), glutathione (GSH), and total nitric oxide (TNO) levels. Total nitric oxide (catalog number K023-H1, Arbor Assays), SOD activity (catalog number 19160-1KT-F, Sigma Aldrich), and glutathione (catalog number CS0260, Sigma Aldrich) were measured in stored serum samples using the colorimetric detection protocol according to manufacturer’s instructions.

### *Ex Vivo* Measurement of Platelet Activation and Intracellular ROS

To further examine the influence of vitamin D on platelet activation, platelets from healthy volunteers were incubated at 37°C in 5% CO_2_ atmosphere in the plasma of healthy control subjects, plasma of T2DM subjects with vitamin D deficiency (baseline), and plasma of T2DM subjects with sufficient vitamin D (6 months). After 1, 3, and 6 h of incubation, the cells were washed and stained with CD62P-APC for 20 min at room temperature. The percentage of P-selectin-positive cells was determined by flow cytometry.

Similarly, to measure the influence of vitamin D on platelet intracellular reactive oxygen species (ROS), platelets from healthy subjects were incubated with different plasma samples from subjects of healthy control, T2DM with vitamin D deficiency, and T2DM with sufficient vitamin D as mentioned above. Furthermore, the cells were washed and stained with DCFDA (catalog number D6883, Sigma Aldrich). The mean fluorescent intensity of DCFDA was assessed using a flow cytometer to measure platelet intracellular ROS.

### Statistical Analysis

Parametric data were presented as mean ± standard deviation, whereas non-parametric data were summarized as median with interquartile range (25th to 75th quartiles). We used Shapiro–Wilk tests to examine the normality of the data. Comparisons between placebo and vitamin D3 groups at baseline were tested using unpaired *t*-test or Mann–Whitney *U*-test, whereas paired Student’s *t*-test or Wilcoxon matched-pair test was used to compare the difference between the baseline and 6-month data. Different heat map visualizations of circulating inflammatory markers were made using the “pheatmap” v1.0.12 package of R programming interface. Box plots were produced using the packages ggplot2 (v0.4.0) and readr (v2.0.2) of R 4.0.2 running under RStudio. The statistical analysis was performed using GraphPad Prism, version 8.0.2 (263) (GraphPad Software, San Diego, CA, USA).

## Results

### Characteristics of the Study Group

A total of 201 patients were screened for the study. A total of 137 participants were excluded from the trial, of whom 134 did not meet the inclusion criteria. Two declined to participate in the study, while one subject was excluded from the study due to access concerns for the subsequent visit. Finally, 64 subjects were enrolled, randomized, and allocated an intervention. Two patients in the vitamin D3 group and three in the placebo group did not attend the follow-up visit. A total of 59 patients [placebo (*n* = 29) and vitamin D3 (*n* = 30)] completed the 6-month follow-up. The baseline characteristics of the participants in each arm are shown in **Table 1**. There was no significant difference observed between the study groups regarding demographic details and clinical and biochemical characteristics. No statistically significant difference was reported in baseline total 25-OH vitamin D levels in the two treatment groups.

**Table 1:** Baseline clinical and biochemical variables in the study groups.

Variables	Study groups	
	Placebo (*n* = 29)	Vitamin D3 (*n* = 30)
Age (years)	55.06 ± 9.57	53.6 ± 9.6
Sex (M/F)	20/9	22/8
Body mass index (kg/m^2^)	26.03 ± 3.28	25.72 ± 4.09
Waist-to-hip ratio	0.97 ± 0.06	0.97 ± 0.07
Systolic BP (mmHg)	137.76 ± 16.47	133.3 ± 15.34
Diastolic BP (mmHg)	82.03 ± 7.63	79.2 ± 7.33
FBS (mg/dl)	191.31 ± 55.49	212.47 ± 63.36
HbA1c (%)	7.93 ± 1.43	8.22 ± 1.30
Total 25-OH vitamin D (ng/ml)	11.95 ± 5.05	14.15 ± 5.8
Duration of T2DM (years)^a^	8 (2–12)	10 (4.75–15)
Total cholesterol (mg/dl)	178.14 ± 32.64	166.7 ± 23.47
Triglycerides (mg/dl)	150.17 ± 51.05	163.43 ± 50.31
HDL (mg/dl)	44.76 ± 8.45	44.07 ± 8.80
LDL (mg/dl)	99.45 ± 19.27	91.4 ± 13.03
Uric acid (mg/dl)	5.94 ± 1.70	5.88 ± 1.38
Creatinine (mg/dl)	1.17 ± 0.26	1.29 ± 0.24
Alcoholic history (yes/no)	5/24	4/26
Smoking history (yes/no)	5/24	6/24
Diabetic medications (%)		
Metformin	3 (10.3)	2 (6.7)
Metformin + sulfonylureas	18 (62.06)	19 (63.3)
Metformin + α-glucosidase inhibitors	2 (6.9)	2 (6.7)
Metformin + DPP-4 inhibitors	1 (3.4)	2 (6.7)
Metformin + sulfonylureas + α-glucosidase inhibitors	5 (17.2)	4 (13.3)
Metformin + sulfonylureas + DPP-4 inhibitors	0 (0)	1 (3.3)

Data are presented as mean ± SD, and unpaired test is used for comparisons between outcome groups.

DPP-4, dipeptidyl peptidase 4.

a Presented as median (Q1–Q3) and compared using Mann–Whitney U-test.

### Serum Vitamin D Metabolite Levels Before and After Intervention

**Figure 2:**
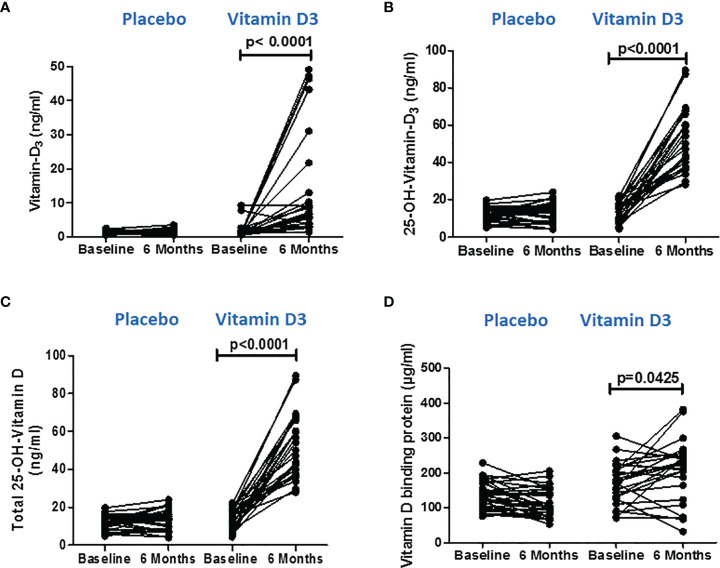
Serum vitamin D metabolite levels after vitamin D supplementation. **(A)** Vitamin D3, **(B)** 25(OH) D3, and **(C)** total 25(OH) D levels at baseline and 6 months in the placebo and vitamin-D3-treated groups. **(D)** Serum vitamin-D-binding protein values at baseline and at 6 months in the placebo and vitamin-D3-treated groups. Wilcoxon matched-pair test is used for the comparison between baseline and 6 months in the placebo and vitamin-D3-treated groups.

We employed LC–MS/MS to assess vitamin D and its metabolites in the serum at baseline and 6 months after the intervention to confirm that vitamin D supplementation increased the serum levels of its metabolites. The results are shown in **Supplementary Table S3**. Vitamin D3 supplementation showed a significant increase in the serum levels of vitamin D3 (baseline, 1.79 ± 0.70; 6 months, 14.17 ± 15.74), 25-OH vitamin D3 (baseline, 14.02 ± 5.76; 6 months, 53.12 ± 16.44), and total 25-OH vitamin D (baseline, 14.15 ± 5.8; 6 months, 51.99 ± 16.46) at 6 months as compared to the baseline levels (*p* < 0.0001). However, no significant changes were observed in the placebo group (**Figures 2A–C**).

Similarly, VDBP, the major regulator of the delivery of vitamin D metabolites to target cells, was measured in serum using the ELISA method. We observed a significant (*p* < 0.05) increase in VDBP level at 6 months (170.6 ± 60.24) of vitamin D supplementation compared to the baseline level (205.4 ± 88.30) (**Figure 2D**).

### Effect of Vitamin D on Glycemic Parameters

**Figure 3:**
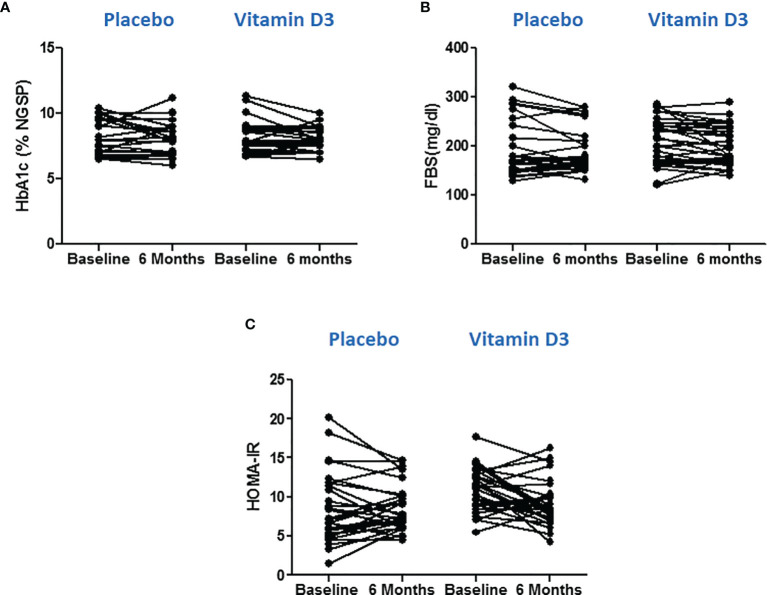
Effect of vitamin D on glycemic parameters. **(A)** Glycated hemoglobin, **(B)** fasting blood sugar, and **(C)** HOMA-IR values at baseline and 6 months in placebo and vitamin D3 treated group. Wilcoxon matched pair test is used for comparison between baseline and 6 months in the placebo and vitamin-D3-teated groups.

Glycemic parameters (FBS and HbA1c) were measured, and HOMA-IR values were calculated to know the effect of vitamin D supplementation on managing type 2 diabetes. However, no significant differences were observed between the values at baseline and at 6 months in the placebo or vitamin D3 group (**Figure 3**).

### Effect of Vitamin D on Immune Cells

Immunome profiling of monocyte cells, natural killer cells, and dendritic cells was done using flow cytometry at baseline and at 6 months of intervention in both the placebo and vitamin D treatment groups. The results are shown in **Table 2**. However, no significant difference was observed in the percentage of different immune cells after the intervention.

**Table 2:** Percentage of immune cells, platelet activation, and platelet–immune cell aggregation in the study groups.

	Placebo (*n* = 29)			Vitamin D3 (*n* = 30)		
	Baseline	6 Months	*P*-value	Baseline	6 months	*P*-value
**Monocyte subsets**						
Classical monocytes (%)	82.44 (76.62–88.36)	78.94 (73.99–83.36)	0.12	84.89 (80.86–87.50)	83.47 (77.58–88.23)	0.76
Intermediate monocytes (%)	9.75 (4.16–12.35)	10.09 (7.62–14.99)	0.07	8.43 (5.80–10.93)	8.49 (6.20–13.55)	0.76
Non-classical monocytes (%)	7.87 (5.13–12.73)	11.79 (9.16–16.30)	0.06	6.93 (4.39–9.11)	8.37 (5.46–11.21)	0.26
**T cell subsets**						
CD4 cells (%)	58.26 (53.29–67.50)	60.12 (49.70–66.31)	0.49	53.14 (49.62–65.37)	58.65 (49.23–67.02)	0.24
CD8 cells (%)	28.69 (20.24–31.06)	26.45 (21.70–32.42)	0.74	26.07 (21.12–33.30)	28.37 (22.40–32.17)	0.94
NKT cells (%)	4.76 (3.59–7.25)	3.75 (2.5–5.30)	0.19	5.17 (3.25–8.35)	3.93 (3.16–5.89)	0.06
NK cells (%)	1.96 (1.30–3.0)	1.32 (0.81–2.37)	0.06	1.98 (1.58–3.08)	1.58 (0.81–3.21)	0.31
**Dendritic cells and subset**						
Dendritic cells (%)	0.33 (0.19–0.43)	0.34 (0.18–0.39)	0.07	0.35 (0.21–0.51)	0.33 (0.20–0.56)	0.74
Myeloid dendritic cells (%)	0.19 (0.12–0.27)	0.13 (0.08–0.24)	0.06	0.24 (0.13–0.37)	0.19 (0.11–0.35)	0.65
Plasmacytoid dendritic cells (%)	0.098 (0.05–0.14)	0.06 (0.03–0.12)	0.26	0.08 (0.05–0.15)	0.08 (0.04–0.16)	1.00
**Platelet activation markers**						
Pac-1 expression (%)	0.29 (0.10–0.51)	0.22 (0.12–0.49)	0.53	0.20 (0.07–0.57)	0.10 (0.09–0.18)	**0.03**
P-selectin expression (%)	56.0 (40.72–63.08)	53.17 (41.45–60.)	0.45	53.83 (42.51–59.76)	34.10 (25.76–47.96)	**<0.001**
**Platelets aggregate formation with innate immune cells**						
Platelet–monocyte aggregates (%)	85.25 (74.33–90.83)	85.35 (76.25–87.33)	0.43	80.0 (71.75–87.337)	49.80 (36.80–67.88)	**<0.001**
Platelet–classical monocyte aggregates (%)	87.55 (73.23–92.96)	84.80 (72.85–86.81)	0.27	84.33 (74.67–89.75)	45.14 (33.11–65.03)	**<0.001**
Platelet–intermediate monocyte aggregates (%)	93.65 (87.11–91.64)	89.92 (83.66–96.95)	0.18	94.80 (88.09–98.12)	72.41 (42.69–87.38)	**<0.001**
Platelet–non-classical monocyte aggregates (%)	92.85 (87.45–95.91)	91.84 (84.73–95.61)	0.96	91.39 (85.44–100)	64.40 (52.73–81.04)	**<0.001**
Platelet–neutrophil aggregates (%)	70.36 (58.60–78.14)	76.01 (59.05–86.83)	0.07	62.96 (54.53–69.92)	54.10 (39.78–65.40)	**0.004**
Platelet–T cell aggregates (%)	25.52 (19.82–33.90)	24.92 (19.69–35.63)	0.39	28.55 (20.91–34.83)	23.25 (19.96–28.66)	**0.001**
Platelet–CD4 cell aggregates (%)	17.90 (12.99–27.16)	19.38 (12.97–29.36)	0.25	20.44 (15.23–30.19)	19.19 (13.85–22.87)	0.07
Platelet–CD8 cell aggregates (%)	28.76 (20.24–34.15)	26.81 (22.23–36.93)	0.82	29.12 (21.12–35.83)	24.36 (21.67–30.22)	0.08
Platelet–NKT cell aggregates (%)	27.52 (18.45–33.77)	30.76 (21.25–37.09)	0.39	29.73 (25.87–36.77)	28.12 (22.04–35.64)	0.48
Platelet–NK cell aggregates (%)	24.55 (18.57–35.50)	19.20 (15.70–29.85)	0.09	26.80 (13.54–33.21)	17.58 (13.24–25.56)	**0.03**
Platelet–dendritic cell aggregates (%)	44.63 (32.33–63.31)	44.85 (35.35–64.25)	0.24	44.11 (34.0–55.89)	31.49 (17.33–48.69)	**0.004**
Platelet–myeloid dendritic aggregates (%)	48.54 (34.91–78.45)	76.66 (50.30–89.20)	0.06	46.86 (26.88–58.70)	39.10 (21.24–70.13)	0.91
Platelet–plasmacytoid dendritic aggregates (%)	43.94 (31.14–51.25)	50.57 (34.13–66.60)	0.23	46.66 (17.39–62.50)	39.53 (27.84–52.72)	0.85

Data are presented as median (25th–75th percentile). Baseline and 6-month intervention data were compared using paired t-test or Wilcoxon rank sum test according to the data distribution. Bold values denote statistical significance p<0.05.

### Effect of Vitamin D Supplementation on Platelet Activation Markers

**Figure 4:**
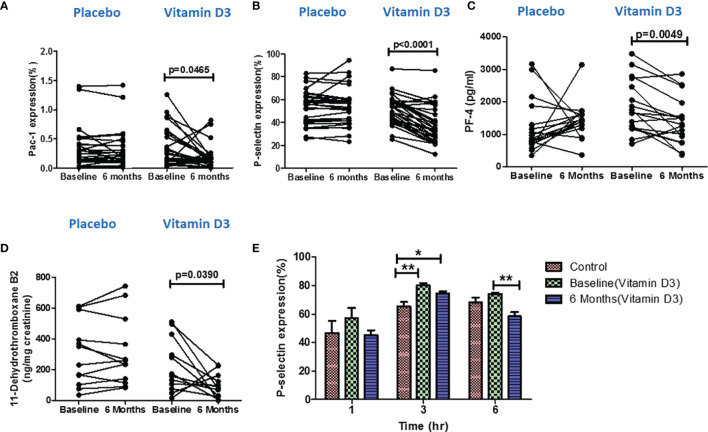
Effect of vitamin D on platelet activation. Percentage of **(A)** PAC-1 expression, **(B)** P-selectin expression, **(C)** platelet factor-4 levels in serum samples, and **(D)** 11-dehydrothromboxane B2 (ng/mg creatinine) level in urine samples at baseline and 6 months in the placebo and vitamin-D3-treated groups. Wilcoxon matched-pair test is used for the comparison between baseline and 6 months in the placebo and vitamin-D3-treated group. **(E)** *Ex vivo* analysis of platelet activation following the incubation of platelets in the plasma samples of healthy control at baseline and 6 months of the vitamin D3 treatment group at different time points (1, 3, and 6 h). One-way ANOVA with Bonferroni test was used for the comparison between outcome groups. * denotes P < 0.05 and ** denotes P < 0.01.

The vitamin D supplementation effects on the expression levels of platelet activation markers (P-selectin and PAC-1) at baseline and at 6 months of placebo and vitamin D are presented in **Figures 4A, B**. Our results showed that vitamin D supplementation significantly reduced both PAC-1 and p-selectin expression in the platelets (*p* < 0.05).

### Effect of Vitamin D Supplementation on the Levels of Circulating Platelet Factor 4

The serum levels of platelet factor 4 (serum marker for platelet activation) were also measured in both placebo and vitamin D treatment groups by the ELISA method. Our result showed that the PF-4 (pg/ml) levels decreased after vitamin D supplementation compared to the baseline levels (**Figure 4C**).

### Effect of Vitamin D Supplementation on the Levels of 11-Dehydrothromboxane B2

We also measured the urinary levels of 11-dehydrothromboxane B2, a urine marker for platelet activation, in both placebo (*n* = 12) and vitamin D treatment (*n* = 12) groups at baseline and at 6 months using the LC–MS/MS technique. We observed a significant decrease in 11-dehydrothromboxane B2 (ng/mg creatinine) levels after 6 months of vitamin D supplementation (**Figure 4D**).

### *Ex Vivo* Assessment of Vitamin D on Platelet Activation

To further confirm the effect of the circulatory levels of vitamin D on platelet activation, we performed an *ex vivo* assay. Platelets that were incubated with the plasma of vitamin-D-sufficient T2DM subjects showed a decreased platelet activation than the platelets incubated in the plasma of vitamin-D-deficient T2DM subjects (**Figure 4E**).

### Effect of Vitamin D on Platelet Aggregate Formation With Immune Cells

**Figure 5:**
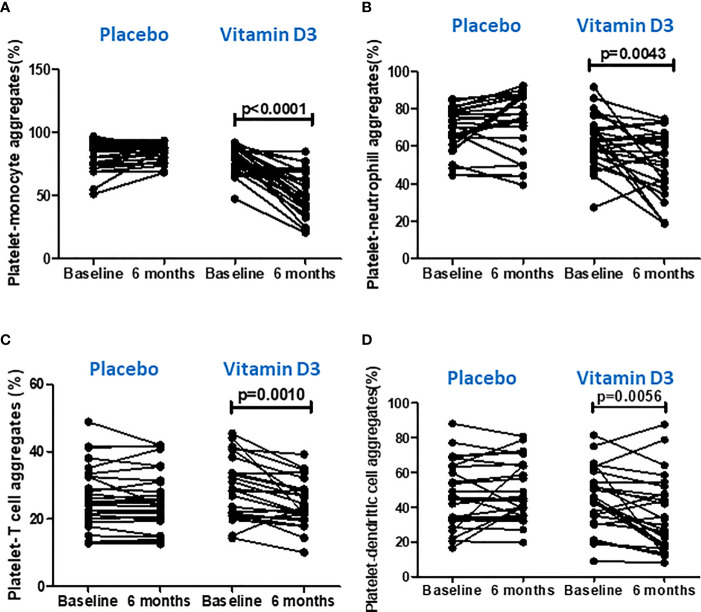
Effect of vitamin D on platelet aggregation with immune cells. **(A)** Platelet–monocyte aggregates, **(B)** platelet–neutrophil aggregates, **(C)** platelet–T cell aggregates, and **(D)** platelet–dendritic cell aggregates at baseline and 6 months in the placebo and vitamin-D3-treated groups. Wilcoxon matched-pair test was used for the comparison between baseline and 6 months in the placebo and vitamin-D3-treated groups.

The vitamin D supplementation effects on platelet–immune cell aggregate formation at baseline and at 6 months of placebo and vitamin D treatment are presented in **Table 2** and **Figure 5**. Platelet aggregate formation with monocytes, classical (CD14++ CD16-) monocytes, intermediate (CD14++ CD16+) monocytes, non-classical (CD14+ CD16++) monocytes, neutrophils, T cells, natural killer (CD3- CD56+) cells, and dendritic (lineage– HLA DR+) cells were significantly reduced after 6 months of vitamin D supplementation. Platelet aggregate with CD4 (CD3+ CD4+) T cells, CD8 (CD3+ CD8+) T cells, natural killer T (CD3+ CD56+) cells, plasmacytoid dendritic (HLA DR+ CD123+) cells, and myeloid dendritic (HLA DR+ CD11C+) cells were also found to be decreased after vitamin D supplementation. However, the changes were not statistically significant. There were no significant changes observed in the placebo group.

### Effect of Vitamin D Supplementation on Circulating Inflammatory Markers

**Figure 6:**
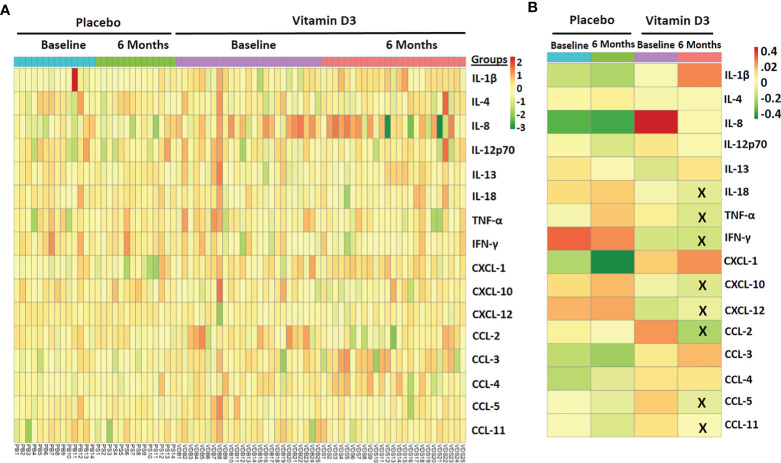
Effect of vitamin D supplementation on circulating inflammatory markers. **(A)** Heat map showing the circulatory inflammatory marker samples at baseline and 6 months in the placebo and vitamin-D3-treated group. **(B)** The median level of circulating markers across different study groups is also shown. The X mark indicates statistically significant change as compared to the baseline level (*p* < 0.05).

**Figure 7:**
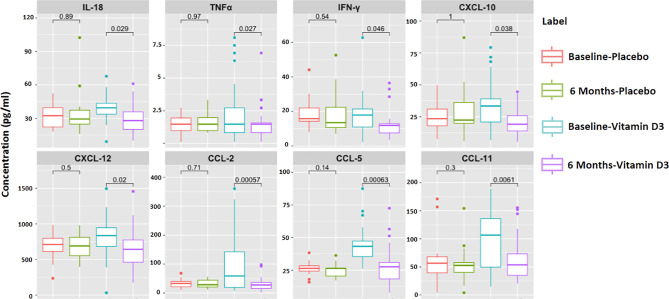
Significantly altered cytokines and chemokines in 6 months of treatment in the placebo and vitamin-D3-treated groups as compared to the baseline level. Wilcoxon matched-pair test is used for the comparison between baseline and 6 months in the placebo and vitamin-D3-treated groups.

A heat map showing the serum levels of the 16 cytokines and chemokines is presented in **Figure 6A**. The median values of each cytokine/chemokine at baseline and at 6 months of the placebo and vitamin D treatment groups are further presented in **Figure 6B**. Our data showed that serum IL-18, TNF-α, IFN-γ, CXCL-10, CXCL-12, CCL-2, CCL-5, and CCL-11 levels significantly decreased after vitamin D supplementation (**Figure 7**). However, no statistically significant changes were observed in the placebo treatment group.

### Effect of Vitamin D on Superoxide Dismutase Activity, Glutathione, and Nitric Oxide Levels

**Figure 8:**
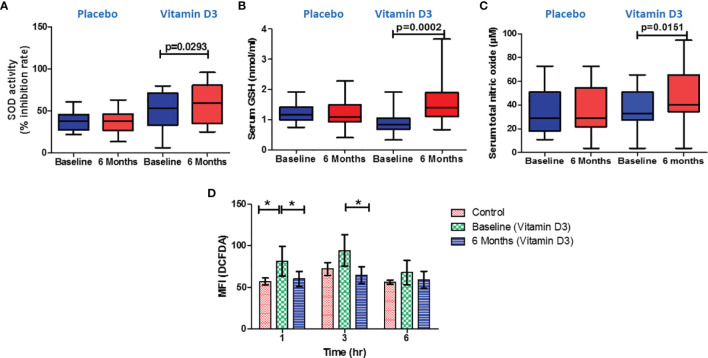
Effect of vitamin D on oxidative stress markers and serum nitric oxide levels. **(A)** Serum superoxide dismutase activity (% inhibition), **(B)** reduced glutathione (nmol/ml), and **(C)** total nitric oxide (µM) at baseline and 6 months in the placebo and vitamin-D3-treated groups. Paired *t*-test or Wilcoxon matched-pair test was used for the comparison between baseline and 6 months in the placebo and vitamin-D3-treated groups. **(D)** *Ex vivo* measurement of intracellular reactive oxygen species in incubated platelets from the plasma samples of healthy control, baseline, and 6 months of the vitamin D3 treatment group at different time points (1, 3, and 6 h). One-way ANOVA with Bonferroni test was used for the comparison between outcome groups. * denotes P < 0.05.

We assessed the SOD activity, glutathione, and total nitric oxide levels in the serum samples to find the mechanism of decreased platelet activation after vitamin D supplementation. We observed increased SOD activity (% inhibition rate) in the serum samples after vitamin D supplementation (*p* < 0.05) (**Figure 8A**). Similarly, the serum levels of glutathione increased after 6 months of vitamin D supplementation (*p* < 0.05) (**Figure 8B**). Total nitric oxide, an inhibitor of platelet activation, was significantly increased in serum samples after vitamin D supplementation (*p* < 0.05) (**Figure 8C**).

### *Ex Vivo* Assessment of Vitamin D on Platelet Intracellular ROS

To confirm the effect of vitamin D on oxidative stress, we have performed an *ex vivo* experiment; platelets incubated in the plasma samples of vitamin-D-sufficient T2DM subjects showed lower intracellular ROS levels than the platelets incubated in the plasma samples of vitamin-D-deficient T2DM subjects (**Figure 8D**).

## Discussion

In the present randomized, placebo-controlled trial, vitamin D3 supplementation improved the circulatory vitamin D levels in type 2 diabetes patients. Our study provides the first and strongest evidence that vitamin D supplementation reduced platelet activation, platelet–immune cell aggregates, inflammation, and oxidative stress in vitamin-D-deficient type 2 diabetes patients. According to previously published literature, vitamin D deficiency is a global health concern, and the prevalence of vitamin D deficiency in India is 80–90% (14). Vitamin D deficiency and the significance of vitamin D in the pathogenesis of type 2 diabetes and associated complications have piqued the researchers’ curiosity. In agreement with previous studies, we observed that vitamin D3 supplementation improved the circulatory levels of vitamin D metabolites. Different doses of vitamin D have been used in clinical trials to treat vitamin D deficiency (15–17). However, our study demonstrates that cholecalciferol (vitamin D3) supplementation with 60,000 IU/week for 3 months, followed by 60,000 IU/month up to 6 months, improved and helped maintain sufficient vitamin D levels in vitamin-D-deficient type 2 diabetes patients. After vitamin D supplementation, the serum levels of total 25-OH vitamin D in all patients were below the toxic levels (<100 ng/ml). Further to this, no clinically significant adverse events were detected in either of the two groups during the research period.

Apart from the vitamin D metabolites, the serum levels of VDBP were also shown to be higher after vitamin D treatment. The vitamin-D-binding protein is a key factor for regulating 25-OH-vitamin D concentrations in the circulation and controls 25-OH vitamin D and 1, 25(OH) _2_ vitamin D bioavailability to target tissues. Similar to our findings, Berg et al. reported increased serum levels of VDBP after vitamin D2 supplementation (18).

The results of the current clinical trial demonstrated that 6 months of vitamin D supplementation did not improve the FBS, HbA1c, or HOMA-IR value. A recent meta-analysis that included 20 randomized controlled trials found that oral vitamin D supplementation did not influence the FBS, HbA1c, and fasting insulin levels in type 2 diabetes (19). Furthermore, another meta-analysis comprised of 22 randomized clinical trials in type 2 diabetes showed a modest 0.32% reduction of HbA1c after vitamin D supplementation (20). However, the effect of vitamin D supplementation on glycemic control in type 2 diabetes is not clear; the difference in ethnicity and genetic background could be the reason behind the variability of observations among the different clinical trials (21).

Our clinical study identified a decreased percentage of PAC-1-positive and P-selectin-positive platelets after the vitamin D intervention. Two individuals in the vitamin D arm had higher PAC-1 expression at six months compared to baseline, which could be attributed to the complexity of the disease state prevalent in T2DM patients. It is reported that insulin resistance, hyperglycemia, inflammation, oxidative stress, and endothelial damage in type 2 diabetes contribute to platelet activation by altering calcium hemostasis, ROS generation, impairing NO release and glycation of platelet proteins (22, 23). Our finding of decreased platelet activation is further confirmed by decreased urine levels of 11-dehydrothromboxane B2 and serum levels of platelet factor 4 after vitamin D supplementation. The 11-dehydrothromboxane B2 is a metabolite of thromboxane A2 and an important urinary marker for platelet activation (24). PF-4 is an inflammatory marker and a circulating platelet activation marker, according to experts (25). Our observation was in agreement with the previous study that vitamin D deficiency contributes to the increased platelet reactivity and platelet aggregation in type 2 diabetes (9, 26).

Our previous research has proven that platelet activation and platelet-immune cells aggregate formation may contribute to inflammation and complexity of both type 2 diabetes and coronary artery disease among type 2 diabetes subjects (3). Hui Min et al. reported that increased platelet-monocyte aggregation in vitamin D deficient healthy subjects (27). It is reported that activated platelets adhere to immune cells and form platelet immune cell aggregation by tethering of platelet surface ligands with their counter receptors on the immune cells (28). In our study, we studied vitamin D effect on platelet-aggregate formation with different immune cells. We observed vitamin D supplementation decreased platelet aggregation with monocytes (and monocyte subsets), neutrophils, T cells, natural killer cells and dendritic cells.

As per available research evidence, the activated platelets release reactive oxygen and nitrogen species, which are critical in developing inflammation and thrombosis (29). Further increased levels of ROS, such as superoxide anion, hydrogen peroxide can directly contribute to platelet activation (30). In the present study we have measured superoxide dismutase (SOD) activity, glutathione (GSH), and total nitric oxide (TNO) to understand the effect of vitamin D on oxidative stress and the mechanism by which vitamin D reduces platelet activation. Following vitamin D administration, we noticed increased superoxide dismutase (anti-oxidant) activity and glutathione levels. Similar to our observation, researchers reported decreased oxidative DNA damage after vitamin D supplementation in patients with metabolic disorders (31). Recently, Imanparast et al. reported that vitamin D3 improves endothelial dysfunction by reducing oxidative stress (32). Nitric oxide is a key marker for endothelial function and also acts as a platelet activation inhibitor (33, 34). Increased nitric oxide after vitamin D intervention may also contribute to reducing the platelet-mediated inflammation in the study subjects. Further, we performed *ex-vivo* experiment to confirm the effect of circulating vitamin D metabolites on platelet activation. We observed decreased activation of healthy platelets when incubated in plasma samples from vitamin D sufficient subjects. Similarly, we observed decreased intracellular ROS in platelets when incubated in vitamin D sufficient plasma as compared to vitamin D deficient plasma of the same subject.

Furthermore, we examined serum cytokines and chemokines to understand the effect of vitamin D supplementation on systemic inflammation in type 2 diabetes. We observed significant decrease in circulating levels of IL-18, TNF-α, IFN-γ, CXCL-10, CXCL-12, CCL-2, CCL-5, and CCL-11. However, we observed a difference in the serum IL-18, IL-8, and IFN-gamma levels between the study groups at baseline. This could be due to the complexity of disease conditions in the study groups. We were more interested in investigating changes in inflammatory markers after vitamin D supplementation. Compared to baseline levels, we observed a significant decrease in IL-18 and IFN-gamma after the vitamin D treatment. No statistically significant difference was observed in most of the clinical and biochemical parameters between the study groups. Apart from this, we have also measured platelet factor 4, and we observed decreased PF-4 levels after vitamin D supplementation in T2DM subjects. It was interesting to know from previous publications that the release of inflammatory mediators like IL-18 (35), TNF-α (36, 37), CXCL-12 (38), PF-4 (39), and CCL-5 (40) can be induced by platelet activation and platelet–immune cell aggregation formation. Moreover, researchers also reported that inflammatory mediators such as IL-18, TNF-α, and PF-4 can significantly contribute to platelet activation in various disease conditions (35, 39, 41). Our findings suggest that decreased platelet activation and platelet immune cell aggregation following vitamin D treatment may reduce systemic inflammation and *vice versa*.

### Limitations

In the present study, we attempted to find the effect of vitamin D supplementation on platelet-mediated inflammation in type 2 diabetes patients. However, our study has a few limitations. The study’s main limitation is being a single-center study design with a small sample size and a short duration. Another limitation of our study is that it does not focus on the molecular mechanism responsible for reducing platelet-mediated inflammation at the cellular level. Further *in vitro* experiments, to evaluate the direct impact of vitamin D on platelets, are required to firmly conclude that vitamin D reduces platelet-mediated inflammation. However, by assessing markers in cells, serum, and urine, our study provides strong evidence that vitamin D reduces platelet-mediated inflammation in type 2 diabetes subjects.

## Conclusion

In conclusion, the correction of vitamin D deficiency in type 2 diabetes patients by vitamin D3 supplementation does not improve the glycemic parameters. Our study highlights that vitamin D supplementation reduces platelet activation, platelet–immune cell aggregates, and platelet-mediated inflammation in type 2 diabetes patients. Our study results provide evidence that cholecalciferol supportive therapy may help to reduce or prevent the disease progression and cardiovascular risk in type 2 diabetes patients. A better understanding of the molecular mechanism behind platelet-mediated inflammation reduction by vitamin D3 needs to be elucidated in future *in vitro* experiments and clinical studies with a large number of type 2 diabetes patients.

## Data Availability Statement

The original contributions presented in the study are included in the article/**Supplementary Material**. Further inquiries can be directed to the corresponding author.

## Ethics Statement

The studies involving human participants were reviewed and approved by Downtown Ethics Committee. The patients/participants provided their written informed consent to participate in this study.

## Author Contributions

EJ and RA conceived and designed the study. EJ, AJ, and BN performed the experiments. Analysis of the data was done by EJ, RB, and RA. Heat map visualizations were made by MJA and EJ. Subject recruitment and clinical characteristic measurement done by RD, IK, and EJ. EJ, RB, and RA wrote the manuscript. All authors contributed to the article and approved the submitted version.

## Conflict of Interest

The authors declare that the research was conducted in the absence of any commercial or financial relationships that could be construed as a potential conflict of interest.

## Publisher’s Note

All claims expressed in this article are solely those of the authors and do not necessarily represent those of their affiliated organizations, or those of the publisher, the editors and the reviewers. Any product that may be evaluated in this article, or claim that may be made by its manufacturer, is not guaranteed or endorsed by the publisher.

## Abbreviations

ACD, acid citrate dextrose; BMI, body mass index; CVD, cardiovascular diseases; DBP, diastolic blood pressure; ELISA, enzyme-linked immunosorbent assay; FBS, fasting blood sugar; HbA1c, glycated hemoglobin; HOMA-IR, Homeostatic Model Assessment of Insulin Resistance; IS, internal standard; IU, international unit; LC–MS/MS, liquid chromatography–mass spectrometry/mass spectrometry; ROS, reactive oxygen species; SBP, systolic blood pressure; SOD, superoxide dismutase; T2DM, type 2 diabetes; VDBP, vitamin D-binding protein.
