# Supplementary material for: Vitamin D Supplementation Modulates Platelet-Mediated Inflammation in Subjects With Type 2 Diabetes: A Randomized, Double-Blind, Placebo-Controlled Trial
Source: Front Immunol. 2022 May 26;13:869591. doi: 10.3389/fimmu.2022.869591 (PMC9205628; doi:10.3389/fimmu.2022.869591)
Supplement: Supplementary file 1 [file DataSheet_1.docx]

**Supplementary File**

**Vitamin D supplementation modulates platelet mediated inflammation in subjects with type 2 diabetes: A randomized, double-blind, placebo-controlled trial**

Ebin Johny, Aishwarya Jala, **Bishamber Nath,** Md Jahangir Alam, Indra Kuladhipati, Rupam Das, Roshan M Borkar, Ramu Adela*

**Study design and dosage pattern of the clinical trial**

^
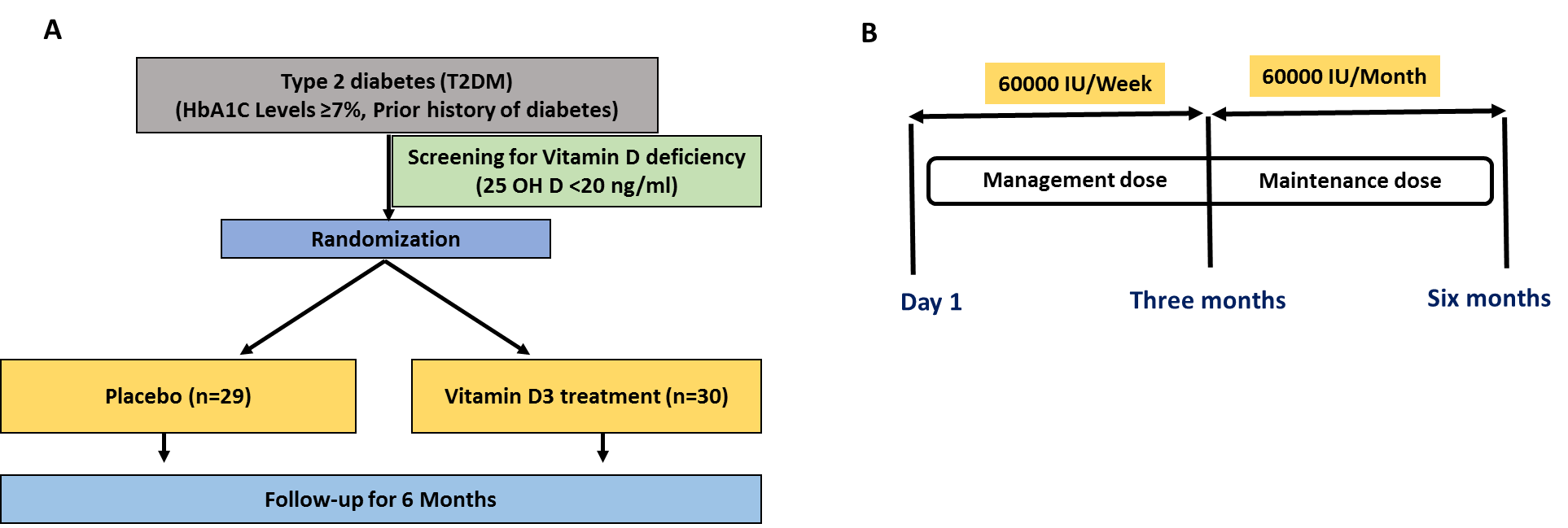
^

**Figure S1:** **A**) Study design **B**) Dosage pattern of the clinical trial

**Quantification of vitamin D metabolites by LC-MS/MS**

Analysis was performed using 1290 Infinity II HPLC equipped with a quaternary pump, a degasser, an autosampler, and a column compartment. Mass spectrometric detection was carried out using a triple quadrupole mass analyzer (Agilent 6495 LC/TQ) equipped with a Jet Stream technology ion source (AJS-ESI) source. The data acquisition was under the control of Mass Hunter workstation (version 10.1). The separation of vitamin D, its metabolites and IS from endogenous substances was achieved using ZORBAX Eclipse Plus C18 Rapid Resolution HD (2.1 X 50mm, 1.8- Micron) and mobile phase consisting of a mixture of 2mM ammonium formate with 0.1% formic acid (A) and methanol with 0.1% formic acid (B) in an gradient program mode. The gradient solvent program was set as follows: (Tmin/% proportion of solvent B): 0/50, 0–0.5/50, 0.5–1/95, 1–21/95, 21–23/50 and 23–25/50. The flow rate of the mobile phase was 0.2 mL/min, the column temperature 45°C, and the injection volume 10μL. The instrument was operated on multiple reaction monitoring mode (MRM). The MRM transitions for vitamin D and its respective metabolites was optimized using MassHunter Optimizer. The source conditions for positive ion AJS-ESI mode were optimized as follows; source gas temperature was set to 250°C, gas flow: 14 l/min, nebulizer 20 psi, sheath gas temperature 250°C, sheath gas flow 11 l/min, capillary voltage +3000V and nozzle voltage 1500V. For quantification, the MRM transitions were set to 397.4>69.1, 385.4>91.1, 413.3>55.2, 401.3>383.3, 446.4>135, 434>399.3, and 399.4>381.4 for vitamin D_2_, vitamin D_3_, 25 (OH) vitamin D_2_, 25 (OH) vitamin D_3_, 1,25 (OH)_2_ vitamin D_2_, 1,25 (OH)_2_ vitamin D_3_ and IS, respectively. The cell accelerator voltage was set to 4V for all the analytes and the collision energies were 36V, 76V, 72V, 4V, 8V, 8V and 20V for vitamin D_2_, vitamin D_3_, 25 (OH) vitamin D_2_, 25 (OH) vitamin D_3_, 1,25 (OH)_2_ vitamin D_2_, 1,25 (OH)_2_ vitamin D_3_ and IS, respectively.

**Method validation for the estimation of vitamin D metabolites by LCMS/MS**

The method developed for the determination of vitamin D and its respective metabolites was validated for determination in control and disease patient samples. The method specificity was confirmed by injecting blank serum (n-=6), corresponding serum spiked with IS (50 ng/mL) and lower limit of quantification (LLOQ). There was no inference at the retention time of analytes and IS. The carry-over effect was also evaluated by injecting the highest concentration and by monitoring the subsequent blank. The results indicated no carry-over effect.

The calibration curve in human serum which was constructed for 6 standards was linear over a range of 5-200 ng/mL for vitamin D_3_, 25 (OH) vitamin D_2_, 25 (OH) vitamin D_3_, 1,25 (OH)_2_ vitamin D_2_, and 1,25 (OH)_2_ vitamin D_3_, and 10-200 ng/mL for vitamin D_2_ in human serum. The relationship of concentration plotted against the analyte/IS ratio was obtained with a weighing factor of 1/X. The correlation coefficient (r^2^) values was found to be > 0.99 for vitamin D and its respective metabolites. The limit of detection vitamin D and its metabolites by this method was 1 ng/mL. The LLOQ of method was 5ng/mL for vitamin D_3_, 25 (OH) vitamin D_2_, 25 (OH) vitamin D_3_, 1,25 (OH)_2_ vitamin D_2_, 1,25 (OH)_2_ vitamin D_3_ and 10ng/mL for vitamin D_2._ The intra-day and inter-day precision and accuracy results vitamin D and its respective metabolites are summarized in **Table S1.** % Recovery and was used to assess the accuracy and % RSD was used to determine the precision of the developed method. The intra-day and inter-day accuracy of vitamin D_2_ and its respective metabolites ranged from 94.9% to 101.86% and 95.75%, to 101.92%, respectively. The inter-day and intraday accuracy of vitamin D_3_ and its respective metabolites ranged from 96.03 to 107.23% and 89.70 to 107.49%, respectively. The intra-day and inter-day precision of vitamin D_2_ and its respective metabolites ranged from 0.66% to 9.07% and 0.53 to 2.81%, respectively. The inter-day and intraday precision of vitamin D_3_ and its respective metabolites ranged from 0.50% to 10.41% and 0.26% to 3.65%, respectively. The data precision and accuracy were found to be within acceptable levels. Matrix effect was assessed at all the QC levels. The results indicated the absence of absolute matrix effect. LC-MS/MS chromatograms for vitamin D and its metabolite acquired in MRM mode was shown in **Figure: S2**.

**Table S1** Intra and inter-day accuracy and precision of vitamin D and it metabolites

| **Analyte** | **QC samples** | **Nominal conc.** | **Intra-day** | | | **Inter-day** | | |
| --- | --- | --- | --- | --- | --- | --- | --- | --- |
|  |  |  | **Calculated conc.** | **Accuracy (%)** | **Precision (%RSD)** | **Calculated conc.** | **Accuracy (%)** | **Precision (%RSD)** |
| **Vitamin D2** | **LLOQ** | 10 | 9.49±0.86 | 94.9±8.61 | 9.07 | 9.57±0.10 | 95.75±1.09 | 1.14 |
|  | **LQC** | 30 | 30.13±0.19 | 100.43±0.65 | 0.66 | 30.21±0.42 | 100.70±1.40 | 1.39 |
|  | **MQC** | 75 | 73.59±0.70 | 98.13±0.93 | 0.95 | 73.53±0.54 | 98.04±0.73 | 0.75 |
|  | **HQC** | 150 | 150.93±1.33 | 100.62±0.88 | 0.88 | 150.09±0.79 | 100.06±0.53 | 0.53 |
| **Vitamin D3** | **LLOQ** | 5 | 4.94±0.17 | 98.93±3.47 | 3.52 | 5.01±0.06 | 100.35±1.23 | 1.23 |
|  | **LQC** | 15 | 14.87±0.23 | 99.18±1.59 | 1.60 | 14.92±0.03 | 99.48±0.26 | 0.26 |
|  | **MQC** | 50 | 48.48±1.81 | 96.96±3.62 | 3.74 | 49.76±1.75 | 99.53±3.48 | 3.50 |
|  | **HQC** | 150 | 150.68±2.22 | 100.45±1.48 | 1.48 | 150.49±1.00 | 100.33±0.67 | 0.67 |
| **25 (OH) Vitamin D3** | **LLOQ** | 5 | 5.36±0.05 | 107.23±1.11 | 1.04 | 5.37±0.04 | 107.49±0.97 | 0.90 |
|  | **LQC** | 15 | 15.25±0.36 | 101.67±2.44 | 2.40 | 15.12±0.12 | 100.48±0.21 | 0.85 |
|  | **MQC** | 50 | 50.68±0.39 | 101.37±0.78 | 0.78 | 50.74±0.10 | 101.48±0.21 | 0.21 |
|  | **HQC** | 150 | 152.45±1.35 | 101.63±0.88 | 0.89 | 152.44±0.19 | 101.63±0.12 | 0.13 |
| **25 (OH) Vitamin D2** | **LLOQ** | 5 | 4.78±1.13 | 95.79±1.08 | 1.13 | 4.86±0.09 | 97.31±1.83 | 1.89 |
|  | **LQC** | 15 | 15.09±0.65 | 100.65±4.34 | 4.31 | 14.79±0.36 | 98.62±2.43 | 2.47 |
|  | **MQC** | 50 | 48.30±1.91 | 96.60±3.82 | 3.96 | 48.75±0.39 | 97.51±0.78 | 0.81 |
|  | **HQC** | 150 | 149.98±1.59 | 99.98±1.60 | 1.06 | 150.10±0.11 | 100.06±0.07 | 0.08 |
| **1,25 (OH)2 Vitamin D3** | **LLOQ** | 5 | 4.35±0.45 | 87.11±9.077 | 10.41 | 4.48±0.16 | 89.7±3.27 | 3.65 |
|  | **LQC** | 15 | 14.40±0.69 | 96.03±4.61 | 4.80 | 14.38±0.10 | 95.88±0.66 | 0.69 |
|  | **MQC** | 50 | 52.61±2.30 | 105.23±4.60 | 4.37 | 51.56±1.03 | 103.13±2.06 | 2.00 |
|  | **HQC** | 150 | 152.79±0.77 | 101.86±0.51 | 0.50 | 152.88±0.24 | 101.92±0.16 | 0.15 |
| **1,25 (OH)2 Vitamin D2** | **LLOQ** | 5 | 5.16±0.28 | 103.33±5.67 | 5.49 | 5.00±0.14 | 100.08±2.81 | 2.81 |
|  | **LQC** | 15 | 14.72±0.57 | 98.15±3.82 | 3.89 | 14.84±0.10 | 95.94±0.68 | 0.68 |
|  | **MQC** | 50 | 50.15±0.77 | 100.30±1.55 | 1.54 | 49.59±0.64 | 99.19±1.28 | 1.29 |
|  | **HQC** | 150 | 152.21±1.28 | 101.47±0.85 | 0.84 | 151.82±0.83 | 101.92±0.55 | 0.54 |


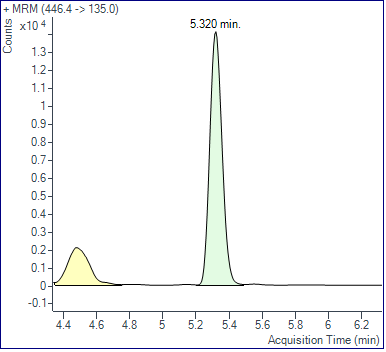

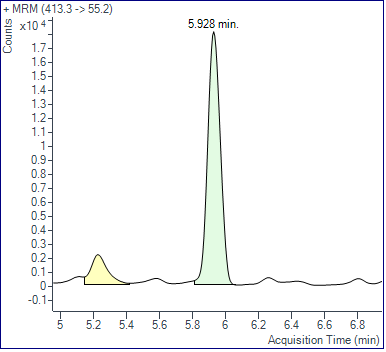

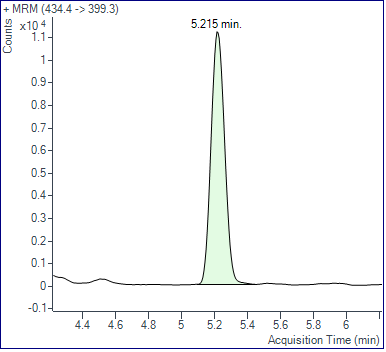

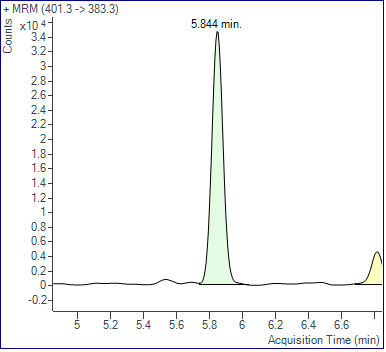

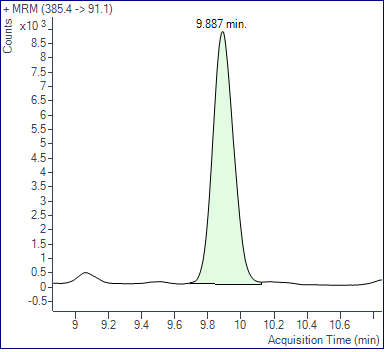

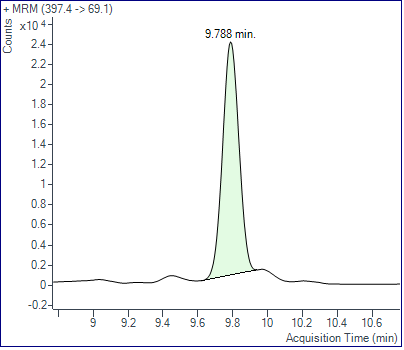

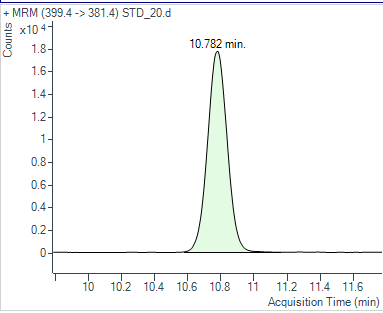


**A**

**B**

**C**

**E**

**F**

**G**

**D**

**Figure S2:**LC-MS/MS chromatograms for vitamin D and its metabolite acquired in MRM mode **A**) vitamin D_2_ **B**) 25 (OH) vitamin D_2_ **C**) 1,25-(OH) _2_ vitamin D_2_  **D**) vitamin D_3_ **E**) 25 (OH)vitamin D_3_ **F**) 1,25-(OH)_2_ **G**) vitamin D_3_ G) IS.

**Gating strategy for the measurement of platelet activation by flowcytometer**

**
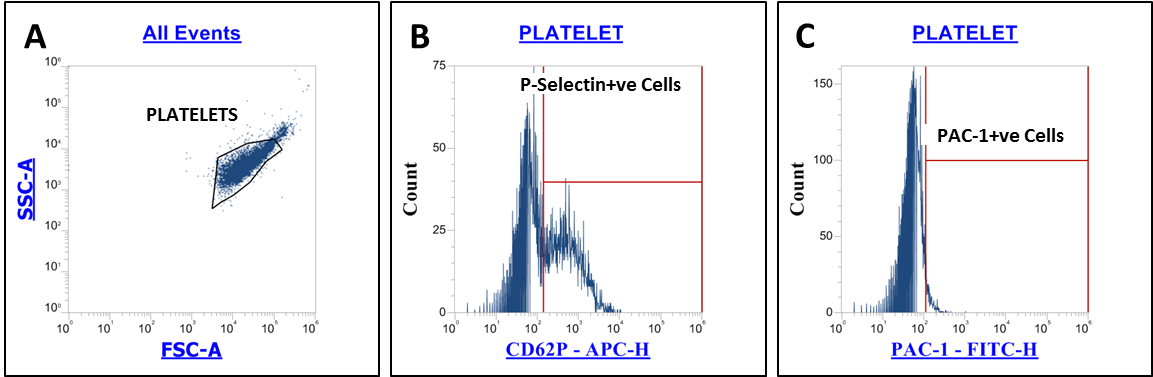
**

**Figure S3:** Measurement of platelets activation. **A**) Identification of platelets based on their forward scatter (FSC) and side scatter characteristic (SSC) from platelet rich plasma. **B**) P-selectin (CD62P) positive and **C**) Pac-1 positive platelets were assessed from the platelet population

**Gating strategy for identification of neutrophils, monocytes and monocyte subsets and platelet-immune cells aggregate by flow cytometer**

**
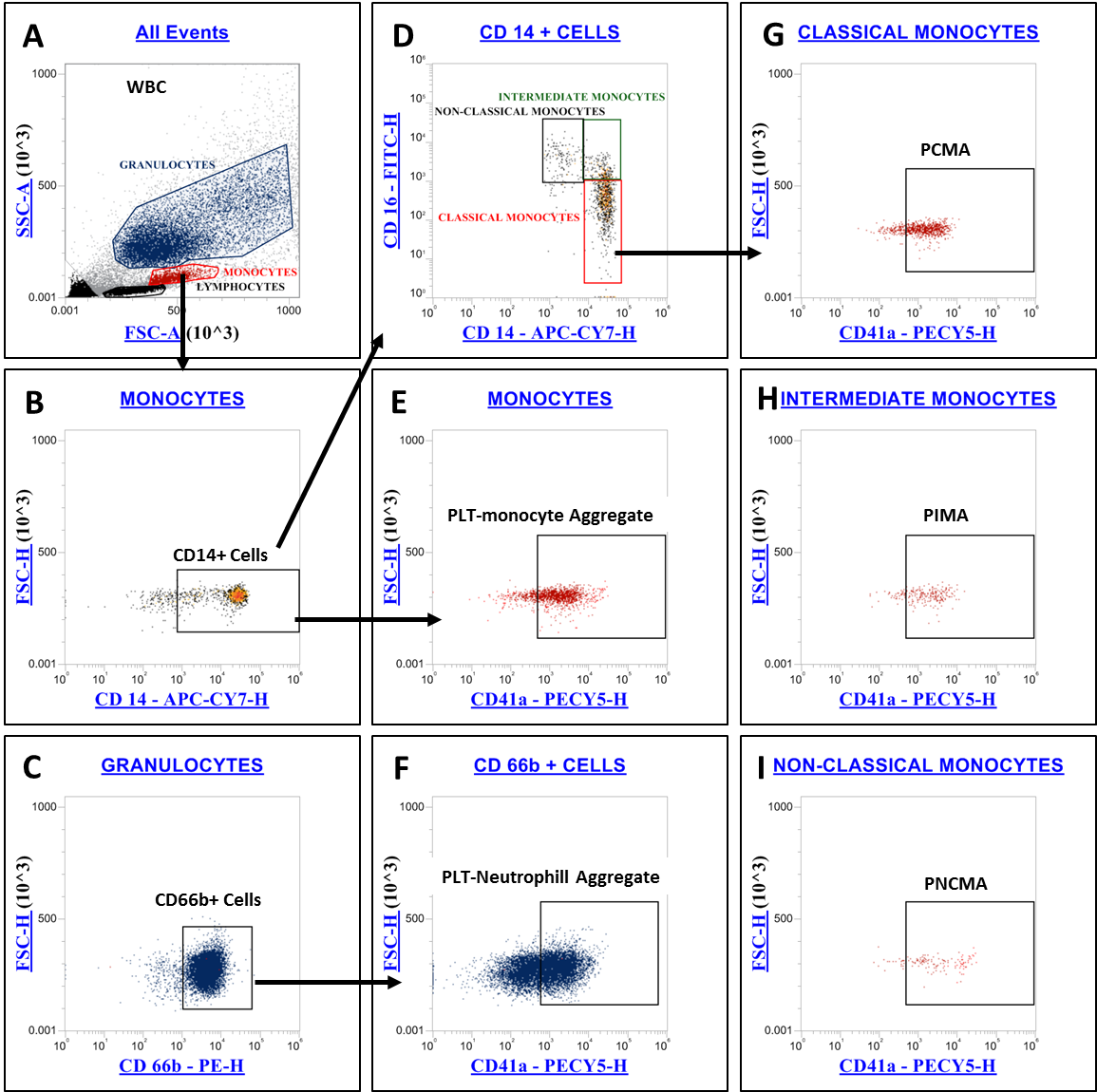
**

**Figure S4:** Identification of neutrophils, monocytes and monocyte subsets and their aggregation with platelet. **A**) Monocytes, granulocytes and lymphocytes were identified based on FSC and SSC characteristics. **B)** Monocytes was confirmed by CD14 expression. **C**) neutrophils was confirmed by CD66b expression **D**) monocytes subsets, classical (CD14++ CD16-), intermediate (CD14++ CD16+) and non-classical (CD14+ CD16++) monocytes. were identified from the CD 14+ cells based on the differential expression of CD 14 and CD 16. Idenitified cells were further checked for the co-expression of CD41a (platelet marker) for detection of **E**) platelet monocyte aggregate **F**) platelet neutrophil aggregate **G**) platelet-classical monocyte aggregate **H**) platelet intermediate monocyte aggregate and **I**) platelet-non classical monocyte aggregate.

**Gating strategy for immunome profiling of lymphocyte, natural killer cells, dendritic cells and identification of their aggregate formation with platelet**

**
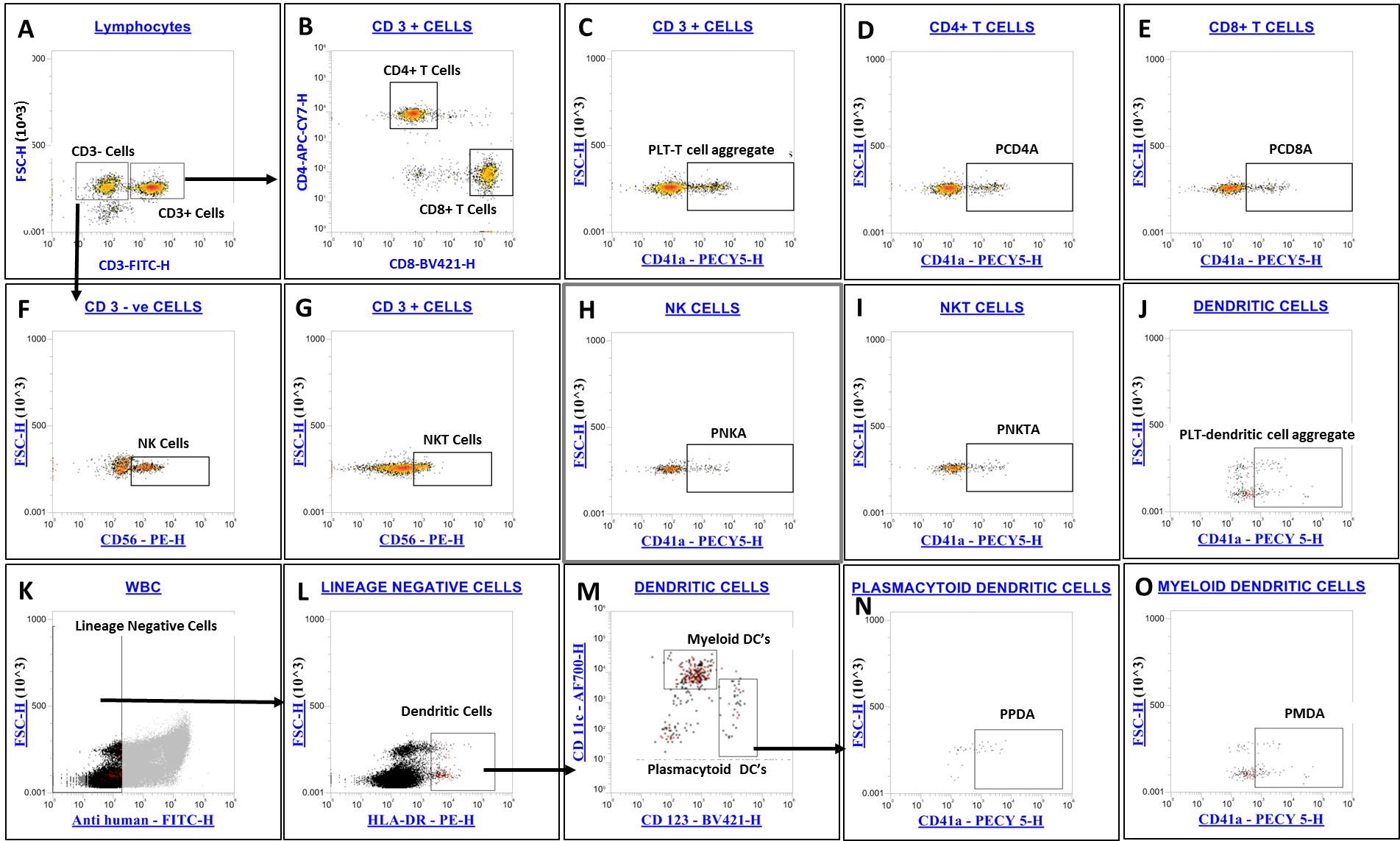
**

**Figure S5:** Immunome profiling of lymphocyte, natural killer cells, dendritic cells and identification of their aggregate formation with platelet **A**) CD3+ve (T cells) and CD3-ve cells were identified from the lymphocyte population. **B**) CD4 T cells (T-helper cells, CD3+ CD4+), CD8 T cells (cytotoxic T cells, CD3+ CD8+) were identified from T cell population. Platelet aggregate formation with **C**) T cells **D**) CD 4 cells and **E**) CD 8 cells was identified.**F**) CD 3- cells were further gated into natural killer cells(CD3-CD56+) and **G**)CD3+ ve cells further gated into natural killer T cells (CD3+CD56+).Platelet aggregate formation with **H**) natural killer cells and **I**) natural killer t cells were determined.**K**)lineage negative cells were gated from the whole WBC cells and gated further into dendritic cells (Lineage-HLA DR+ve). **M**) Dendritic cells further gated into myeloid((HLA DR+ CD11C+) and plasmacytoid (HLA DR+ CD123+) dendritic cells. Platelet aggregate formation with J) dendritic cells N) plasmacytoid dendritic cells and O) myeloid dendritic cells were also identified.

**Gating strategy using fluorescence minus one (FMO) control method for** **identification of platelet-immune cell aggregate formation**


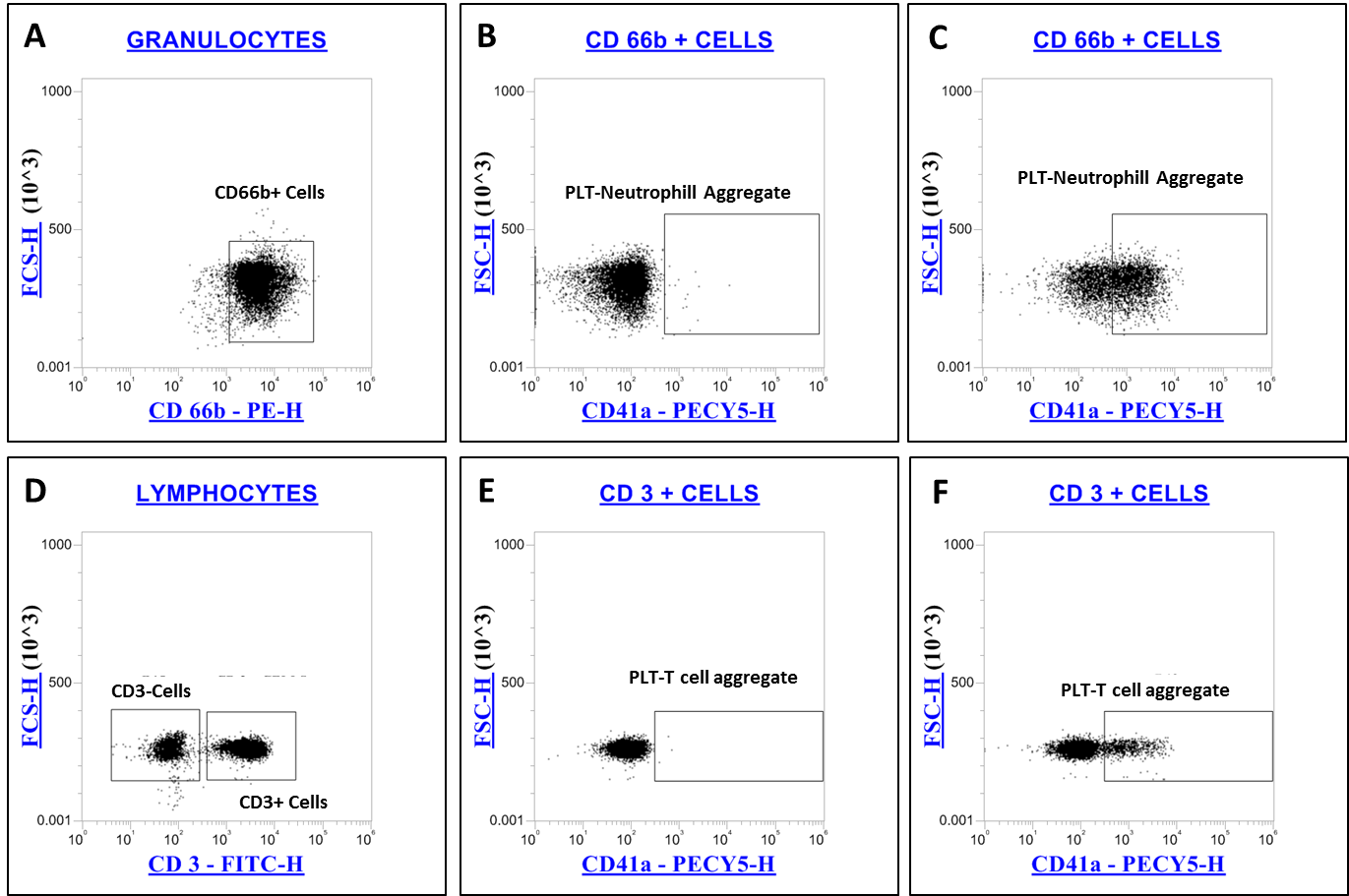


**Figure S6:** Identification of platelet-immune cell aggregate formation using FMO method. **A)** Neutrophils was confirmed by CD66b expression. **B)** FMO control (CD41a PECY5 negative) for identification of platelet-neutrophil aggregates. **C)** Identification of platelet-neutrophill aggregates. **D**) CD3+ve (T cells) and CD3-ve cells were identified from the lymphocyte population. **E)** FMO control (CD41a PECY5 negative) for identification of platelet-T cell aggregates. **F)** Identification of platelet-T cell aggregates.

**Method development and validation for the estimation of 11-dehydrothromboxane B2 in urine by LCMS/MS**

**Preparation of standards**

The stock solution of 11-dehydroythromboxane B2 was prepared in methanol. Further, working solutions for calibration curve (0.5-250 ng/mL) were prepared in methanol from the primary stock solutions. Similarly, internal standard (IS) i.e., 11-dehydrothromboxane B2-d4 of 50 ng/mL was prepared.

**Extraction of 11 11-dehydrothromboxane B2 from urine samples**

An aliquot of 500 µl of urine was spiked with 50 µl of internal standard to make a final concentration of 50 ng/mL. Agilent Bond Elut C18 SPE cartridges were used for the extraction of the analyte. The cartridges were preconditioned with 1mL methanol and equilibrated with 1mL water. The sample was loaded and washed with 1ml of water followed by 2 ml of water-methanol (95:5) mixture and hexane. The analyte was eluted from the cartridge using 2 mL of ethyl acetate. The eluate was dried under vacuum using Eppendorf concentrator plus. The sample is reconstituted in 100 µl of acetonitrile: water mixture (10:90) and subjected to LC-MS/MS analysis.

**Instrumentation**

Analysis was performed using 1290 Infinity II HPLC coupled with triple quadrupole mass analyzer (Agilent 6495 LC/TQ) equipped with a Jet Stream technology ion source (AJS-ESI) source. The separation of 11-dehydroythromboxane B2 and IS from endogenous substances was achieved using ZORBAX Eclipse Plus C18 Rapid Resolution HD (2.1 X 50mm, 1.8- Micron) and mobile phase consisting of a mixture of 2mM ammonium formate with 0.1% formic acid (A) and methanol with 0.1% formic acid (B) in an gradient program mode. The gradient solvent program was set as follows: (Tmin/% proportion of solvent B): 0-0.2/2, 0.2-5/95, 5-8/95, 8-10/2 and 10-14/2. The flow rate of the mobile phase was 0.2 mL/min, the column temperature 45°C, and the injection volume 3 μL. The instrument was operated on multiple reaction monitoring mode (MRM). The MRM transitions for 11-dehydroythromboxane B2 was optimized using MassHunter Optimizer. The source conditions for negative ion AJS-ESI mode were optimized as follows source gas temperature was set to 250°C, gas flow: 14 l/min, nebulizer 20 psi, sheath gas temperature 250°C, sheath gas flow 11 l/min, capillary voltage 3000V and nozzle voltage 1500V. For quantification, the MRM transitions were set to 367.2>161.2, and 371.2>309.2, for 11-dehydroythromboxane B2 and IS, respectively. The cell accelerator voltage was set to 4V for the analytes and the collision energies were 20V, and 16V for 11-dehydroythromboxane B2 and IS, respectively.

**Method validation**

The method developed for the determination of 11-dehydroythromboxane B2 in urine was validated for determination in control and disease patient samples. The method specificity was confirmed by injecting blank urine (n-=6), corresponding urine spiked with IS (50 ng/mL) and lower limit of quantification (LLOQ). There was no inference at the retention time of analyte and IS. The carry-over effect was also evaluated by injecting the highest concentration and by monitoring the subsequent blank. The results indicated no carry-over effect.

The calibration curve in human urine which was constructed for two standards was linear over a range of 0.5-200 ng/mL for 11-dehydroythromboxane B2. The relationship of concentration plotted against the analyte/IS ratio was obtained with a weighing factor of 1/X. The correlation coefficient (r^2^) values was found to be 0.999 for 11 dehydroythromboxane B2. The limit of detection 11-dehydroythromboxane B2 by this method was 0.25 ng/mL. The LLOQ of method was 0.5 ng/mL for the analyte_._ The intra-day and inter-day precision and accuracy results 11-dehydroythromboxane B2 are summarized in **Table S2**. The data precision and accuracy were found to be within acceptable levels. Matrix effect was assessed at all the QC levels. The results indicated the absence of absolute matrix effect

**Table S2** Method validation result for determination of 11-dehydrothromboxane B2

| **Analytes** | **QC samples** |  | **Intra-day** | | | **Inter-day** | | |
| --- | --- | --- | --- | --- | --- | --- | --- | --- |
|  |  | **Nominal conc.** | **Calculated conc.** | **Accuracy (%)** | **Precision (%RSD)** | **Calculated conc.** | **Accuracy (%)** | **Precision (%RSD)** |
| **11-dehydro thromboxane B2** | **LLOQ** | 0.5 | 0.45±0.07 | 91.57±16.86 | 16.86 | 0.46±0.01 | 92.40±3.96 | 4.29 |
|  | **LQC** | 1.5 | 1.52±0.07 | 102.39±4.58 | 4.58 | 1.44±0.07 | 96.59±3.96 | 5.21 |
|  | **MQC** | 10 | 10.39±0.15 | 103.98±1.49 | 1.50 | 10.30±0.08 | 103.04±0.88 | 0.86 |
|  | **HQC** | 100 | 100.90±1.11 | 100.90±1.11 | 1.10 | 101.55±0.57 | 101.55±0.57 | 0.57 |


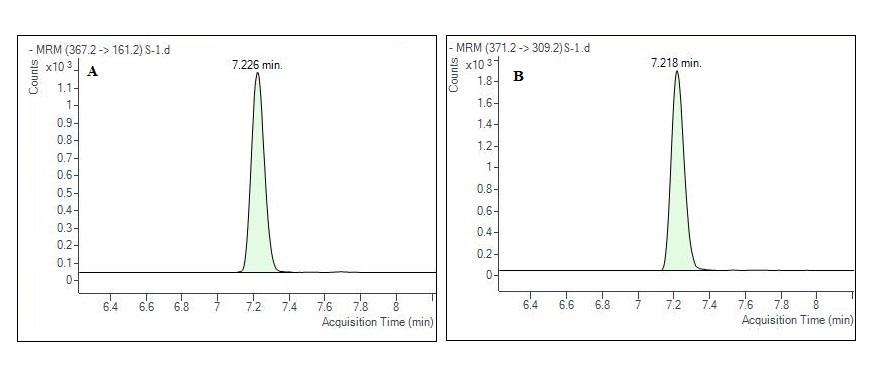


**Figure S7:** LC-MS/MS chromatograms for **A**) 11-dehydrothromboxane B2 and **B**) [11-dehydrothromboxane B2-d4](https://pubchem.ncbi.nlm.nih.gov/compound/24778496)

| **[Vitamin D metabolites](https://pubchem.ncbi.nlm.nih.gov/compound/24778496)** | **[Placebo (n=29)](https://pubchem.ncbi.nlm.nih.gov/compound/24778496)** | | | **[Vitamin D3 (n=30)](https://pubchem.ncbi.nlm.nih.gov/compound/24778496)** | | |
| --- | --- | --- | --- | --- | --- | --- |
|  | **[Baseline](https://pubchem.ncbi.nlm.nih.gov/compound/24778496)** | **[6 Months](https://pubchem.ncbi.nlm.nih.gov/compound/24778496)** | **[P Value](https://pubchem.ncbi.nlm.nih.gov/compound/24778496)** | **[Baseline](https://pubchem.ncbi.nlm.nih.gov/compound/24778496)** | **[6 Months](https://pubchem.ncbi.nlm.nih.gov/compound/24778496)** | **[P value](https://pubchem.ncbi.nlm.nih.gov/compound/24778496)** |
| [Vitamin D3(ng/ml)](https://pubchem.ncbi.nlm.nih.gov/compound/24778496) | [1.25 ± 0.53](https://pubchem.ncbi.nlm.nih.gov/compound/24778496) | [1.51±0.70](https://pubchem.ncbi.nlm.nih.gov/compound/24778496) | [0.07](https://pubchem.ncbi.nlm.nih.gov/compound/24778496) | [1.79±0.70](https://pubchem.ncbi.nlm.nih.gov/compound/24778496) | [14.17±15.74](https://pubchem.ncbi.nlm.nih.gov/compound/24778496) | [<0.0001](https://pubchem.ncbi.nlm.nih.gov/compound/24778496) |
| [25-OH-vitamin D3(ng/ml)](https://pubchem.ncbi.nlm.nih.gov/compound/24778496) | [11.79 ± 5.04](https://pubchem.ncbi.nlm.nih.gov/compound/24778496) | [13.30±6.20](https://pubchem.ncbi.nlm.nih.gov/compound/24778496) | [0.07](https://pubchem.ncbi.nlm.nih.gov/compound/24778496) | [14.02±5.76](https://pubchem.ncbi.nlm.nih.gov/compound/24778496) | [51.99±16.44](https://pubchem.ncbi.nlm.nih.gov/compound/24778496) | [<0.0001](https://pubchem.ncbi.nlm.nih.gov/compound/24778496) |
| [1,25-(OH)](https://pubchem.ncbi.nlm.nih.gov/compound/24778496)_[2](https://pubchem.ncbi.nlm.nih.gov/compound/24778496)_[-vitamin D3(ng/ml)](https://pubchem.ncbi.nlm.nih.gov/compound/24778496) | [0.20 ± 0.48](https://pubchem.ncbi.nlm.nih.gov/compound/24778496) | [0.013±0.04](https://pubchem.ncbi.nlm.nih.gov/compound/24778496) | [0.07](https://pubchem.ncbi.nlm.nih.gov/compound/24778496) | [0.02 ± 0.10](https://pubchem.ncbi.nlm.nih.gov/compound/24778496) | [0.12±0.52](https://pubchem.ncbi.nlm.nih.gov/compound/24778496) | [0.57](https://pubchem.ncbi.nlm.nih.gov/compound/24778496) |
| [Vitamin D2(ng/ml)](https://pubchem.ncbi.nlm.nih.gov/compound/24778496) | [0.011 ± 0.05](https://pubchem.ncbi.nlm.nih.gov/compound/24778496) | [0.07 ± 0.22](https://pubchem.ncbi.nlm.nih.gov/compound/24778496) | [0.31](https://pubchem.ncbi.nlm.nih.gov/compound/24778496) | [0.29±0.71](https://pubchem.ncbi.nlm.nih.gov/compound/24778496) | [0.28±0.43](https://pubchem.ncbi.nlm.nih.gov/compound/24778496) | [0.81](https://pubchem.ncbi.nlm.nih.gov/compound/24778496) |
| [25-OH-vitamin D2(ng/ml)](https://pubchem.ncbi.nlm.nih.gov/compound/24778496) | [0.16 ± 0.84](https://pubchem.ncbi.nlm.nih.gov/compound/24778496) | [0.22 ± 1.06](https://pubchem.ncbi.nlm.nih.gov/compound/24778496) | [0.25](https://pubchem.ncbi.nlm.nih.gov/compound/24778496) | [0.11 ± 0.37](https://pubchem.ncbi.nlm.nih.gov/compound/24778496) | [0.03 ± 0.15](https://pubchem.ncbi.nlm.nih.gov/compound/24778496) | [0.25](https://pubchem.ncbi.nlm.nih.gov/compound/24778496) |
| [1,25-(OH)](https://pubchem.ncbi.nlm.nih.gov/compound/24778496)_[2](https://pubchem.ncbi.nlm.nih.gov/compound/24778496)_ [vitaminD2(ng/ml)](https://pubchem.ncbi.nlm.nih.gov/compound/24778496) | [0.40 ± 0.02](https://pubchem.ncbi.nlm.nih.gov/compound/24778496) | [0.41 ± 0.02](https://pubchem.ncbi.nlm.nih.gov/compound/24778496) | [0.33](https://pubchem.ncbi.nlm.nih.gov/compound/24778496) | [0.41 ± 0.03](https://pubchem.ncbi.nlm.nih.gov/compound/24778496) | [0.40 ± 0.02](https://pubchem.ncbi.nlm.nih.gov/compound/24778496) | [0.07](https://pubchem.ncbi.nlm.nih.gov/compound/24778496) |
| [Total 25-OH D(ng/ml)](https://pubchem.ncbi.nlm.nih.gov/compound/24778496) | [11.95 ± 5.05](https://pubchem.ncbi.nlm.nih.gov/compound/24778496) | [13.53 ± 6.13](https://pubchem.ncbi.nlm.nih.gov/compound/24778496) | [0.06](https://pubchem.ncbi.nlm.nih.gov/compound/24778496) | [14.15 ±5.8](https://pubchem.ncbi.nlm.nih.gov/compound/24778496) | [53.12.±16.46](https://pubchem.ncbi.nlm.nih.gov/compound/24778496) | [<0.0001](https://pubchem.ncbi.nlm.nih.gov/compound/24778496) |
| [Total 1,25-(OH)](https://pubchem.ncbi.nlm.nih.gov/compound/24778496)_[2](https://pubchem.ncbi.nlm.nih.gov/compound/24778496)_ [vitamin D(ng/ml)](https://pubchem.ncbi.nlm.nih.gov/compound/24778496) | [0.56±0.43](https://pubchem.ncbi.nlm.nih.gov/compound/24778496) | [0.41±0.05](https://pubchem.ncbi.nlm.nih.gov/compound/24778496) | [0.93](https://pubchem.ncbi.nlm.nih.gov/compound/24778496) | [0.44±0.12](https://pubchem.ncbi.nlm.nih.gov/compound/24778496) | [0.47±0.52](https://pubchem.ncbi.nlm.nih.gov/compound/24778496) | [0.24](https://pubchem.ncbi.nlm.nih.gov/compound/24778496) |

**[Table S3](https://pubchem.ncbi.nlm.nih.gov/compound/24778496)** [Vitamin D metabolites levels in the study groups](https://pubchem.ncbi.nlm.nih.gov/compound/24778496)

**[Notes:](https://pubchem.ncbi.nlm.nih.gov/compound/24778496)** [Data represented as Mean ± SD. Baseline and 6 months intervention data was compared by using paired t test or Wilcoxon rank test according to the data distribution. p<0.05 was considered significant.](https://pubchem.ncbi.nlm.nih.gov/compound/24778496)
